# CuCo_2_S_4_ Nanoparticles Embedded in Carbon Nanotube Networks as Sulfur Hosts for High Performance Lithium-Sulfur Batteries

**DOI:** 10.3390/nano12183104

**Published:** 2022-09-07

**Authors:** Hongying Wang, Yanli Song, Yanming Zhao, Yan Zhao, Zhifeng Wang

**Affiliations:** 1 School of Materials Science and Engineering, Hebei University of Technology, Tianjin 300401, China; 2 Key Laboratory for New Type of Functional Materials in Hebei Province, Hebei University of Technology, Tianjin 300401, China

**Keywords:** CuCo_2_S_4_, nanoparticles, carbon nanotubes, sulfur host, lithium-sulfur batteries

## Abstract

Rational design of sulfur hosts for lithium-sulfur (Li-S) batteries is essential to address the shuttle effect and accelerate reaction kinetics. Herein, the composites of bimetallic sulfide CuCo_2_S_4_ loaded on carbon nanotubes (CNTs) are prepared by hydrothermal method. By regulating the loading of CuCo_2_S_4_ nanoparticles, it is found that when Cu^2+^ and CNT are prepared in a 10:1 ratio, the CuCo_2_S_4_ nanoparticles loaded on the CNT are relatively uniformly distributed, avoiding the occurrence of agglomeration, which improves the electrical conductivity and number of active sites. Through a series of electrochemical performance tests, the S/CuCo_2_S_4_-1/CNT presents a discharge specific capacity of 1021 mAh g^−1^ at 0.2 C after 100 cycles, showing good cycling stability. Even at 1 C, the S/CuCo_2_S_4_-1/CNT cathode delivers a discharge capacity of 627 mAh g^−1^ after 500 cycles. This study offers a promising strategy for the design of bimetallic sulfide-based sulfur hosts in Li-S batteries.

## 1. Introduction

Lithium-sulfur (Li-S) batteries are the up-and-coming next-generation rechargeable batteries because of the merits of being environment-friendly, their high energy density (2600 Wh kg^−1^) and theoretical capacity (1675 mAh g^−1^) [1,2,3]. However, soluble lithium polysulfides (LiPSs) are dissolved into the electrolyte during the charge–discharge process, which induces the shuttle effect and rapid capacity decay, limiting the exploitation of high-performance Li-S batteries [4,5,6,7,8]. Therefore, various solutions, including the design of sulfur host, separator and electrolyte modification, are committed to solving the above problem. Among them, the design and preparation of suitable sulfur carriers play an essential role in boosting the performance of Li-S batteries.

In previous studies, various carbon materials including carbon spheres, carbon nanofibers and carbon nanotubes (CNTs) were used as sulfur hosts in Li-S batteries by virtue of physical adsorbing LiPSs. This method presents the effect of sulfur fixation to a certain extent; however, it still has some limitations [9,10,11,12]. Some studies reported that polar materials including metal oxides, metal sulfides and metal phosphides, etc., could mitigate the shuttle effect effectively by chemical adsorption and catalysis [11,12,13,14], such as SiO_2_ [15], MnO_2_/TiO_2_ [16], nickel-plated [17] and CoP-CNT@C [18]. Among them, transition metal sulfides not only interact strongly with LiPSs but also show excellent catalytic activity in the electrochemical reaction. In addition, it can stabilize the electrochemical performance and enhance the energy efficiency of Li-S batteries [19,20]. For example, CoS_2_ [21,22], NiS [23,24] and Co_3_S_4_ [25,26] were reported to improve the electrochemical performance by a synergistic role of adsorption and catalysis. Compared to monometallic sulfides, bimetallic sulfides possess lower band gap energy and improved electrical conductivity [27]. Simultaneously, bimetallic sulfides can provide more reactive sites than monometallic sulfides. Therefore, extensive research has been devoted to the development of new bimetallic sulfides catalyst. Huang et al. prepared Co-Fe bimetallic sulfides with robust chemical adsorption and catalytic activity, it exhibited a high reversible capacity of 1126.5 mAh g^−1^ at 0.2 C [28]. Lu et al. fabricated the NiCo_2_S_4_@CNTs/S for Li-S batteries. CNTs were found to promote the electronic transportation capacity and conductivity of the cathode material effectively, while NiCo_2_S_4_ showed strong adsorption toward the LiPSs, effectively suppressing the diffusion of LiPSs [29]. Previous work has proved that bimetallic sulfide/carbon composite can show a strong effect in inhibiting the shuttle effect. However, the development of different polysulfide/carbon composites is still lacking at present, and the conductivity, electrochemical stability, and conversion kinetics need to be further improved.

In this work, CNTs loaded with CuCo_2_S_4_ bimetallic sulfides (CuCo_2_S_4_/CNT) were prepared and used as the sulfur host for Li-S batteries. By further regulating the loading amount of CuCo_2_S_4_ nanoparticles on CNT materials, it is explored that the appropriate loading amount of CuCo_2_S_4_ nanoparticles can effectively improve the kinetics of LiPSs conversion, inducing a good electrochemical performance. The as-obtained S/CuCo_2_S_4_-1/CNT can sustain a specific capacity of 627 mAh g^−1^ after 500 cycles, with a capacity decay rate of only 0.08% per cycle.

## 2. Materials and Methods

### Fabrication of CuCo_2_S_4_/CNT and CuCo_2_S_4_

A total of 15 mg slightly oxidized carbon nanotubes were ultrasonically dispersed into 30 mL ethylene glycol, and the suspension was sonicated for 2 h with stirring. Then, 0.15 g Cu(CH_3_COO)_2_-H_2_O (A reagent) and 0.0265 g Co(CH_3_COO)_2_-4H_2_O (B reagent) were dissolved in the mixture and stirred magnetically for 1 h. Afterwards, 0.117 g thiourea was added and stirred for 40 min. The mixture was poured into a 50 mL autoclave, sealed and reacted at 180 °C for 24 h. After cooling, the mixture was cleaned by centrifugation with anhydrous ethanol four times. The product was gathered and dried under vacuum at 70 °C to obtain CuCo_2_S_4_-1/CNT. Holding all other parameters constant, CuCo_2_S_4_-2/CNT was also obtained by adding 0.3 g A reagent and 0.053 g B reagent, while CuCo_2_S_4_-0.5/CNT can be obtained by adding 0.075 g A reagent and 0.01325 g B reagent. CuCo_2_S_4_ nanoparticles were obtained without adding slightly oxidized carbon nanotubes and ethylene glycol under the same fabrication conditions as CuCo_2_S_4_/CNT above.

Further details about the fabrication of the S/CuCo_2_S_4_/CNT and S/CuCo_2_S_4_ composites, preparation of Li_2_S_6_ solution, material characterization, electrochemical measurements and symmetric cells measurement, can be obtained from Supporting S0.

## 3. Results and Discussion

**Figure 1:**
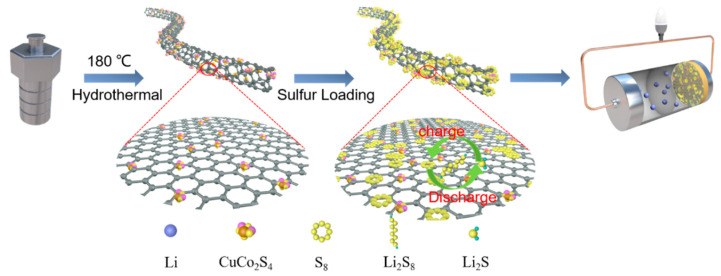
Schematic diagram showing the synthesis and structure of S/CuCo_2_S_4_/CNT.

**Figure 2:**
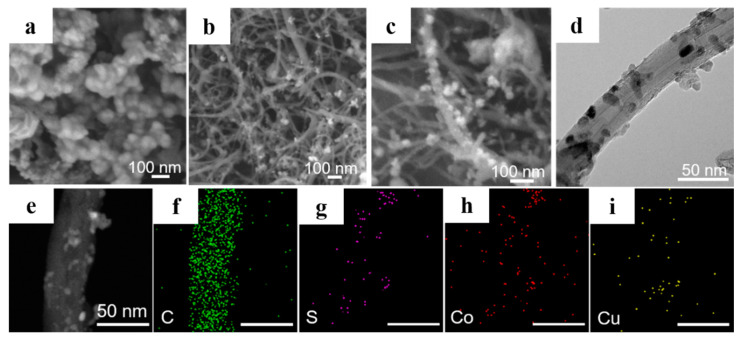
SEM images of (**a**) CuCo_2_S_4_, (**b**,**c**) CuCo_2_S_4_-1/CNT; (**d**) TEM images of CuCo_2_S_4_-1/CNT. (**e**) TEM image of CuCo_2_S_4_-1/CNT and the corresponding elemental mappings: (**f**) C, (**g**) S, (**h**) Co, (**i**) Cu.

The schematic of the synthesis process and structure of S/CuCo_2_S_4_/CNT is shown in Figure 1. In brief, CuCo_2_S_4_/CNT is first synthesized by the hydrothermal method. Then, S/CuCo_2_S_4_/CNT can be obtained by heating of S and CuCo_2_S_4_/CNT mixture. The detailed process can be found in Supporting S0. The final product S/CuCo_2_S_4_/CNT was used as a cathode in this work for Li-S batteries application. By adjusting the content of Cu(CH_3_COO)_2_-H_2_O and Co(CH_3_COO)_2_-4H_2_O, the ratio of CuCo_2_S_4_ particles loaded on CNTs can be regulated. The products are marked as CuCo_2_S_4_-0.5/CNT, CuCo_2_S_4_-1/CNT and CuCo_2_S_4_-2/CNT, respectively, with the increase in contents of raw materials. As shown in Figure 2a, when CuCo_2_S_4_ particles were synthesized by hydrothermal method, the particle size was about 30–55 nm. However, severe particle agglomeration occurs which reduces the specific surface area of the material. As shown in Figure S1, although the loading of CuCo_2_S_4_ on CNT (CuCo_2_S_4_-0.5/CNT) inhibits CuCo_2_S_4_ agglomeration, the loading is too sparse (Figure S1a), which limits the adsorption ability toward polysulfides. While the loading of CuCo_2_S_4_ on CNTs is too dense for CuCo_2_S_4_-2/CNT (Figure S1c), restraining the exposure of active sites. The scanning electron microscope (SEM) images of CuCo_2_S_4_-1/CNT (Figure 2b,c and Figure S1b) exhibits uniform loading of CuCo_2_S_4_ particles on the CNTs’ surface, which most possibly enhances the performance of Li-S batteries. Transmission electron microscope (TEM) images of CuCo_2_S_4_-1/CNT in Figure 2d also confirm that CNTs are closely covered by CuCo_2_S_4_ with a granular diameter of 8–15 nm. Furthermore, it can be found from the above images that the CNTs are multi-walled. The average diameter and lengths of CNTs are 34 nm and 2 μm, respectively. In addition, when CNTs are exposed to air, they are inevitably oxidized. Some oxygen-containing groups, such as epoxide (C−O−C), hydroxyl (−OH), carboxyl (−COOH), and carbonyl (C=O), may be produced on the CNTs’ surface [30]. The presence of these oxygen-containing groups may affect the loading of CuCo_2_S_4_, as well as the electrochemical performance of Li-S batteries. Therefore, related tests need to be further explored in the future. The corresponding element mapping demonstrates the uniform distribution of S, Co, Cu (CuCo_2_S_4_ particle) on CNTs (Figure 2e–i).

**Figure 3:**
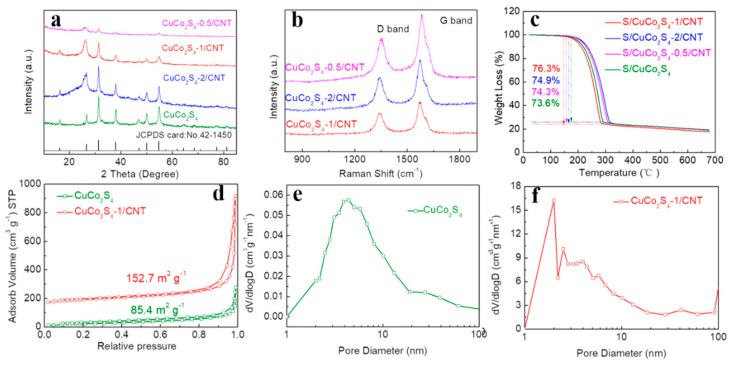
(**a**) XRD patterns of experimental materials. (**b**) Raman spectrum of CuCo_2_S_4_-0.5/CNT, CuCo_2_S_4_-1/CNT and CuCo_2_S_4_-2/CNT. (**c**) TGA plots of S/CuCo_2_S_4_, S/CuCo_2_S_4_-0.5/CNT, S/CuCo_2_S_4_-1/CNT and S/CuCo_2_S_4_-2/CNT. (**d**) N_2_ adsorption/desorption isotherms of CuCo_2_S_4_ and CuCo_2_S_4_-1/CNT. Pore size distribution of (**e**) CuCo_2_S_4_ and (**f**) CuCo_2_S_4_-1/CNT.

The crystal structures of CuCo_2_S_4_, CuCo_2_S_4_-0.5/CNT, CuCo_2_S_4_-1/CNT and CuCo_2_S_4_-2/CNT materials were characterized by X-ray diffraction (XRD) (Figure 3a). The XRD patterns of four samples exhibit five characteristic diffraction peaks at 26.4°, 31.3°, 38.0°, 50.2° and 54.9°, matching with (220), (311), (400), (511) and (440) planes of CuCo_2_S_4_ (JCPDS 42–1450), respectively. The Raman spectra of CuCo_2_S_4_-0.5/CNT, CuCo_2_S_4_-1/CNT and CuCo_2_S_4_-2/CNT samples are shown in Figure 3b. The obvious peak near 1353 cm^−1^ can be marked as the D peak reflecting disordered and defective carbon, while the peak at 1587 cm^−1^ is attributed to the G peak of carbon, relating to the presence of sp^2^-hybridized carbon. The intensity ratio of D peak to G peak of CuCo_2_S_4_-1/CNT (I_D_/I_G_, 0.69) is lowest in the experimental materials, indicating that the graphitization degree and electric conductivity of CuCo_2_S_4_-1/CNT are higher than that of CuCo_2_S_4_-0.5/CNT (0.81), CuCo_2_S_4_-2/CNT (0.77) and CNT (0.85) (Figure S2) [31,32]. In addition, Figure 3c displays the thermogravimetric analysis (TGA) plots of different composites. It could be seen that S/CuCo_2_S_4_-1/CNT presents higher sulfur loading up to 76.3%. The specific surface area and pore size characteristics of CuCo_2_S_4_-1/CNT, CuCo_2_S_4_-2/CNT, CuCo_2_S_4_-0.5/CNT and CuCo_2_S_4_ were studied by N_2_ adsorption-desorption experiments (Figure 3d and Figure S3a,c). It displays typical type III isotherms with H3 type hysteresis loop, indicating the existence of mesopores. CuCo_2_S_4_-1/CNT (152.7 m^2^ g^−1^) shows a higher surface area than CuCo_2_S_4_-2/CNT (138.6 m^2^ g^−1^), CuCo_2_S_4_-0.5/CNT (102.5 m^2^ g^−1^) and CuCo_2_S_4_ (85.4 m^2^ g^−1^). Pore distribution reveals that there exists a large proportion of micropores in CuCo_2_S_4_-1/CNT compared with the other three materials (Figure 3e,f and Figure S3b,d). This is beneficial to enhance the sulfur limitation by physical role. Higher surface area also facilitates the exposure of active sites and provides a rich electrode/electrolyte interface for LiPSs conversion.

**Figure 4:**
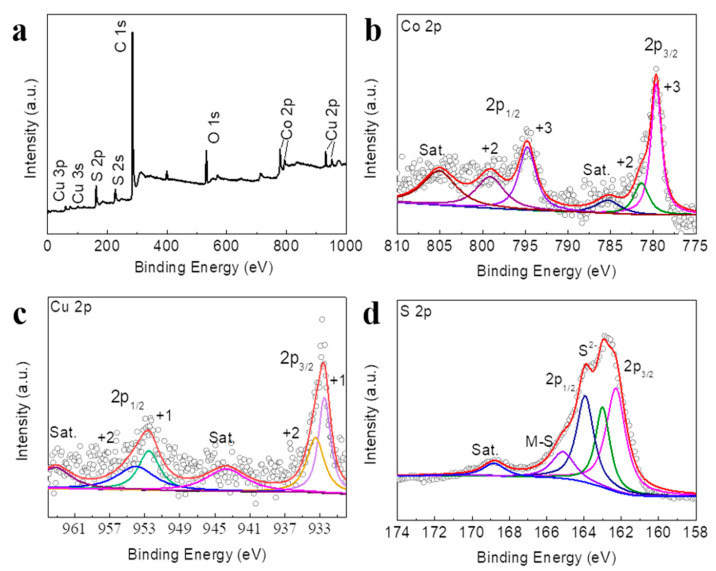
XPS spectra of CuCo_2_S_4_-1/CNT (**a**) Survey; (**b**) Co 2p; (**c**) Cu 2p; and (**d**) S 2p.

In Li-S batteries, X-ray photoelectron spectrometry (XPS) is usually used to determine the composition, structure and element content of the material. Therefore, in order to identify the composition and valence of the CuCo_2_S_4_-1/CNT, we conducted XPS measurement. It can be concluded that Cu, Co, S, C, and O elements exist in CuCo_2_S_4_-1/CNT (Figure 4a). The Co XPS spectrum (Figure 4b) shows six peaks at 794.8 eV for Co^3+^ 2p_1/2_, 779.5 eV for Co^3+^ 2p_3/2_, 798.8 eV for Co^2+^ 2p_1/2_, 781.4 eV for Co^2+^ 2p_3/2_, 805.1 and 785.2 eV for satellite peaks [33]. In the Cu XPS spectrum (Figure 4c), the binding energy values at 952.5 eV and 932.5 eV correspond to Cu^+^ 2p_1/2_ and Cu^+^ 2p_3/2_, respectively. While 954.0 eV and 933.5 eV can be contributed to Cu^2+^ 2p_1/2_ and Cu^2+^ 2p_3/2_, and 943.7 eV and 963.2 eV for satellite peaks [34]. In addition, two characteristic peaks in the S 2p XPS spectra at 163.9 eV (2p_1/2_) and 162.2 eV (2p_3/2_) correspond to S^2^^−^species (Figure 4d) [35,36]. The lower intensity characteristic peak at 168.8 eV suggests the presence of small amounts of sulphate or sulfite species and the presence of thin oxide layers on the surface. The peak at 165.1 eV probably corresponds to an M-S bond (M = Cu or Co), where the sulfur presents in the form of polysulfides (S_n_^2−^, 2 ≤ n < 8) [37]. In addition, the present type of polysulfides in different charge–discharge states can be detected by XPS, which can provide a better understanding of the charge–discharge mechanism of lithium-sulfur batteries. These in-depth analyses and discussions will be carried out and published in the future.

In order to investigate the feasibility of S/CuCo_2_S_4_/CNT composites as Li-S batteries cathodes, a series of electrochemical performance tests were carried out. As shown in Figure S4, the red lines and blue lines correspond to the standard PDF cards of sulfur (JCPDS 08-0247) and CuCo_2_S_4_ (JCPDS 42-1450), respectively. The XRD results of S/CuCo_2_S_4_/CNT composites also show characteristic diffraction peaks of S and CuCo_2_S_4_, indicating a successful sulfur loading. The final mass ratios of CuCo_2_S_4_ to CNT in S/CuCo_2_S_4_-0.5/CNT, S/CuCo_2_S_4_-1/CNT, and S/CuCo_2_S_4_-2/CNT composites are calculated by combining XPS, EDS and inductively coupled plasma mass spectrometry (ICP-MS) results, showing 4.92:1, 9.81:1 and 18.53:1, respectively, which are close to the theoretical materials input ratios of 5:1, 10:1 and 20:1.

**Figure 5:**
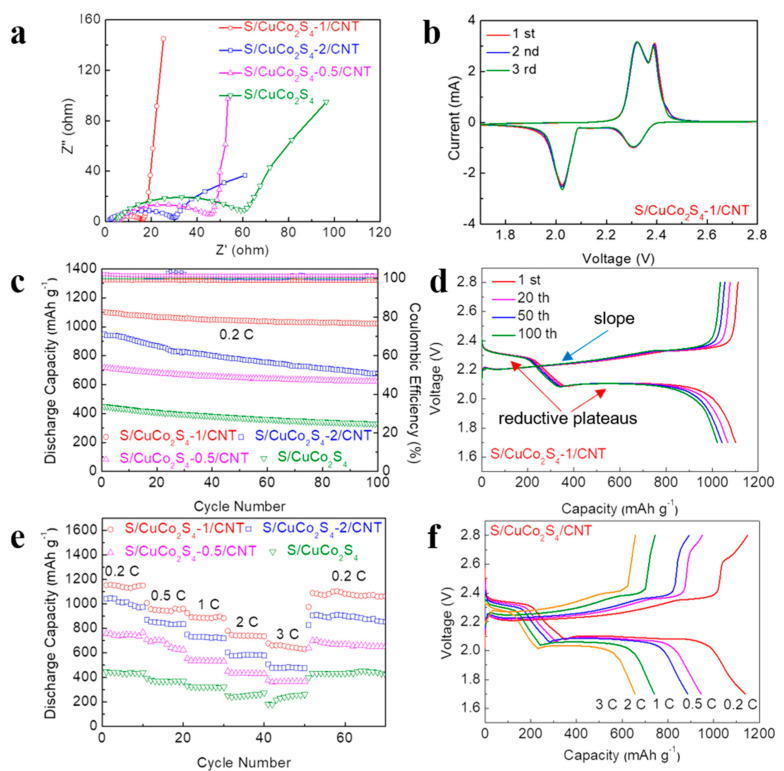
(**a**) Nyquist plots of S/CuCo_2_S_4_, S/CuCo_2_S_4_-0.5/CNT, S/CuCo_2_S_4_-1/CNT and S/CuCo_2_S_4_-2/CNT cathodes before cycling. (**b**) CV curves at the scan rate of 0.1 mV s^−1^ of S/CuCo_2_S_4_-1/CNT cathodes. (**c**) Cycling performances of S/CuCo_2_S_4_, S/CuCo_2_S_4_-0.5/CNT, S/CuCo_2_S_4_-1/CNT and S/CuCo_2_S_4_-2/CNT cathodes at 0.2 C. (**d**) Charge/discharge voltage profiles of S/CuCo_2_S_4_-1/CNT at 0.2 C. (**e**) Rate performances of S/CuCo_2_S_4_, S/CuCo_2_S_4_-0.5/CNT, S/CuCo_2_S_4_-1/CNT and S/CuCo_2_S_4_-2/CNT cathodes. (**f**) Charge/discharge voltage profiles at 0.2 C, 0.5 C, 1 C, 2 C and 3 C of S/CuCo_2_S_4_-1/CNT.

Figure 5a shows the Nyquist plots of Li-S batteries of different cathodes. The electrochemical impedance spectroscopy (EIS) curves contain a semicircle and a slope line, in line with the charge transfer resistance and the Warburg bulk impedance, respectively. The charge-transfer resistance of S/CuCo_2_S_4_-1/CNT is smaller than other electrodes, indicating it has the smallest charge-transfer resistance [38]. As shown in Figure 5b, the cyclic voltammetry (CV) curves at 0.1 mV s^−1^ show two distinct reduction peaks during discharge at 2.02 V and 2.31 V. The reduction peak at 2.31 V represents the reduction of S_8_ to soluble LiPSs (Li_2_S_n_, n = 4, 6, 8). The peak at 2.02 V represents the conversion reaction of LiPSs to Li_2_S_2_/Li_2_S. During charging, the oxidation peak splits into two peaks, which are attributed to the oxidation from solid Li_2_S to LiPSs and eventually to S_8_ [39,40]. Furthermore, the first three cycles of CV curves of the S/CuCo_2_S_4_-1/CNT composite are well overlapped, reflecting excellent cycle reversibility. In addition, the first cycle CV curves of S/CuCo_2_S_4_-1/CNT, S/CuCo_2_S_4_-2/CNT and S/CuCo_2_S_4_-0.5/CNT cathodes at the scan rate of 0.1 mV s^−1^ are shown in Figure S5. It is obvious that S/CuCo_2_S_4_-1/CNT has the largest current response, indicating that it has superior catalytic performance. At a low current, the charging–discharging process of Li-S battery is relatively slow, it tends to produce more LiPSs, which dissolve in the electrolyte, causing the shuttle effect. In this way, we can verify the limitation of the shuttle effect by different types of CuCo_2_S_4_ and CNT composites [41]. Moreover, a lot of works have also examined electrochemical performance at 0.2 C so that we can fully compare the electrochemical data of this work with previously published works. Therefore, we perform measurements at 0.2 C based on the above considerations. Figure 5c shows the cycling property of different materials at 0.2 C. The S/CuCo_2_S_4_-1/CNT cathode shows the best electrochemical performance with a first discharge capacity of 1104.5 mAh g^−1^ and a very low cycle decay rate. In fact, each type was prepared for three samples. One battery of S/CuCo_2_S_4_-1/CNT presents an initial capacity of 1364.5 mAh g^−1^. While the other two samples of S/CuCo_2_S_4_-1/CNT cathode show the first discharge capacity of 1100.3 mAh g^−1^ and 1108.9 mAh g^−1^ at 0.2 C (Figure S6). Considering that one of the values is abnormally high, we conservatively choose the other two similar values to report. Therefore, the average discharge capacity with an error is 1104.6 ± 4.3 mAh g^−1^. After 100 cycles, it can maintain a high cycle capacity (1021 mAh g^−1^) and its coulomb efficiency closes to 100%, demonstrating the excellent reversibility of the reaction. In contrast, the S/CuCo_2_S_4_-0.5/CNT and S/CuCo_2_S_4_-2/CNT cathodes exhibited rapid capacity decay and low cycling capacity. In addition, we also compare the S/CuCo_2_S_4_ samples without CNT, which exhibit the lowest cycling performance. This can be attributed to the fact that it lacks the CNT’s hollow structure and three-dimensional conducting framework. For charge–discharge curves of different samples (Figure 5d and Figure S7), there are two obvious reductive plateaus and a slope, which are related to the reduction and oxidation of LiPSs. The voltage profiles of the S/CuCo_2_S_4_-1/CNT cathode exhibit slower capacity decay and smaller polarization, demonstrating it has excellent catalytic activity. The rate performance of different electrode materials is exhibited in Figure 5e. The specific discharge capacities of S/CuCo_2_S_4_-1/CNT at 0.2, 0.5, 1, 2 and 3 C are 1138 mAh g^−1^, 943 mAh g^−1^, 887 mAh g^−1^, 741 mAh g^−1^ and 656 mAh g^−1^, respectively, which is higher than the other three electrode materials. Even when the current density reverts to 0.2 C, the capacity of S/CuCo_2_S_4_-1/CNT can reach 1072 mAh g^−1^, demonstrating the efficient and reversible use of the active sulfur. Moreover, the charge–discharge curves of S/CuCo_2_S_4_-1/CNT at different current densities (Figure 5f) can maintain the characteristic discharge plateau of Li-S batteries compared with S/CuCo_2_S_4_-0.5/CNT, S/CuCo_2_S_4_-2/CNT and S/CuCo_2_S_4_ (Figure S8) [42].

**Figure 6:**
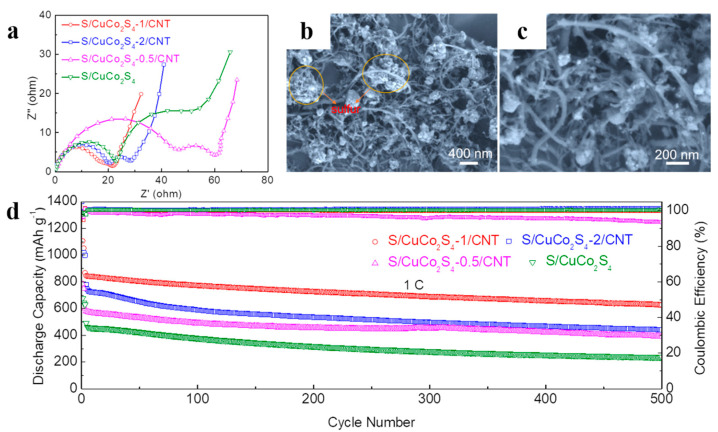
(**a**) Nyquist plots of S/CuCo_2_S_4_, S/CuCo_2_S_4_-0.5/CNT, S/CuCo_2_S_4_-1/CNT and S/CuCo_2_S_4_-2/CNT cathodes after 100 cycles. (**b**,**c**) SEM image of S/CuCo_2_S_4_-1/CNT cathodes after charge–discharge cycle at 0.2 C. (**d**) Cycling performances of S/CuCo_2_S_4_, S/CuCo_2_S_4_-0.5/CNT, S/CuCo_2_S_4_-1/CNT and S/CuCo_2_S_4_-2/CNT cathodes at 1 C.

To further investigate the effect of S/CuCo_2_S_4_-1/CNT on the electrochemical performance, we also carried out the EIS test and morphology analysis after cycling for 100 cycles. As shown in Figure 6a, the impedance diagram is composed of two semicircles and an oblique line. The first semicircle represents the formation of the Li_2_S_2_–Li_2_S interface (R_SEI_). It can be concluded that S/CuCo_2_S_4_-1/CNT has the lowest impedance, indicating its superior electrochemical kinetics [43,44]. Moreover, the morphology of CuCo_2_S_4_-1/CNT after cycling remains relatively intact. The carbon nanotubes retain their original conductive skeleton structure (Figure 6b,c). Based on the above results, long-term cycling performance at 1 C was also carried out. As exhibited in Figure 6d, the specific capacity of S/CuCo_2_S_4_-1/CNT can maintain at 627 mAh g^−1^ after 500 cycles, and the capacity decay rate is only 0.08%/cycle. In contrast, S/CuCo_2_S_4_-2/CNT, S/CuCo_2_S_4_-0.5/CNT and S/CuCo_2_S_4_ decayed to 441, 389 and 236 mAh g^−1^ after 500 cycles, respectively. This can be ascribed to the good catalytic effect of the CuCo_2_S_4_-1/CNT composite on the conversion of LiPSs.

**Figure 7:**
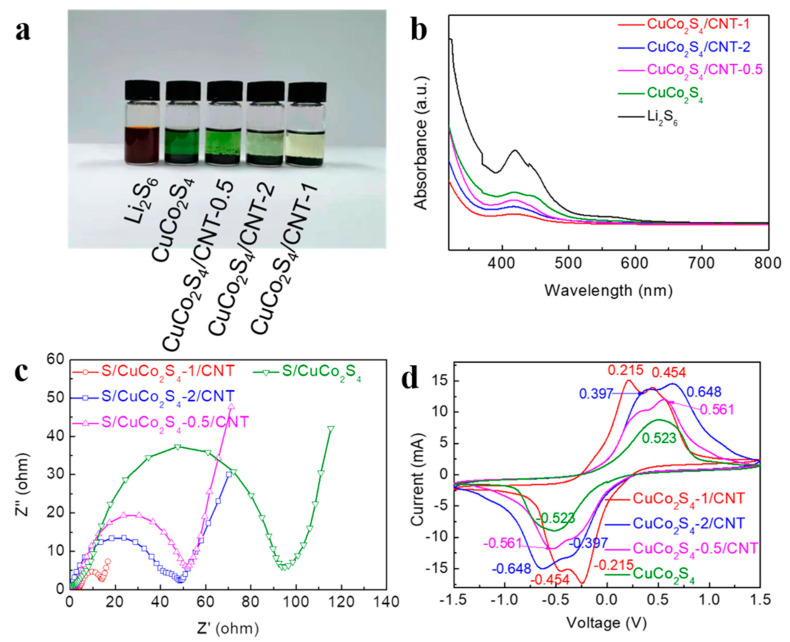
(**a**) Optical images and (**b**) UV-vis spectra after LiPSs adsorption by S/CuCo_2_S_4_-1/CNT, S/CuCo_2_S_4_-2/CNT, S/CuCo_2_S_4_-0.5/CNT and S/CuCo_2_S_4_. (**c**) EIS spectra and (**d**) CV curves at 6 mV s^−1^ of symmetric cells with CuCo_2_S_4_-1/CNT, CuCo_2_S_4_-2/CNT, CuCo_2_S_4_-0.5/CNT and CuCo_2_S_4_ electrodes.

In order to explore its potential mechanism in improving the electrochemical performance of Li-S batteries, the adsorption experiments were performed firstly by immersing the different materials in Li_2_S_6_ solution. Equal amounts of samples of CuCo_2_S_4_-1/CNT, CuCo_2_S_4_-2/CNT, CuCo_2_S_4_-0.5/CNT and CuCo_2_S_4_ were added to the same volume of Li_2_S_6_ solution, and the mixed solutions stand for 24 h. Then, as shown in Figure 7a, the Li_2_S_6_ solution with CuCo_2_-1/CNT material became clear, demonstrating the significant adsorption effect of CuCo_2_S_4_-1/CNT material. Simultaneously, the ultraviolet-visible (UV-Vis) spectrum also confirms the results (Figure 7b) [45,46]. In addition, to further investigate the electrocatalytic performance, symmetric cells were also assembled toward different materials. In Figure 7c, the EIS curve shows that the CuCo_2_S_4_-1/CNT electrode has the lowest resistance, confirming its excellent electrochemical reaction kinetics. The CV curves of the CuCo_2_S_4_-1/CNT electrode clearly show the sharpest redox peaks at −0.215/0.215 V and −0.454/0.454 V and the smallest polarization, proving the most excellent catalyzing behavior of the LiPSs conversion (Figure 7d). In addition, as shown in Figure S9, the first three cycles of CV curves of the CuCo_2_S_4_-1/CNT electrode have a relatively high degree of overlap, demonstrating relatively good reversibility [47,48]. Based on the above electrochemical data, CNTs improve the overall conductivity of composites and promote efficient ion/electron transport. At the same time, the highly interconnected 3D conductive network frameworks provide adequate space to buffer volume changes during the charging–discharging cycle. In addition, the uniform loading of CuCo_2_S_4_ particles on CNTs surface guarantee abundant active sites on CuCo_2_S_4_-1/CNT, which further ensures that the material possesses a high loading of active sulfur. The CuCo_2_S_4_-1/CNT composite presents strong adsorption and catalytic conversion ability for LiPSs. In conclusion, the excellent electrochemical performance of the S/CuCo_2_S_4_-1/CNT cathode can be attributed to the synergistic effect of CuCo_2_S_4_ and CNTs.

## 4. Conclusions

In summary, bimetallic sulfide CuCo_2_S_4_ nanoparticles loaded with CNT composites were synthesized by the hydrothermal method in this work. By modulating the different loadings of the CuCo_2_S_4_ nanoparticles, it is found that the CuCo_2_S_4_-1/CNT composites effectively improved the property of Li-S batteries, which can be attributed to the improved overall electrical conductivity of the CNT, promoting efficient ion/electron transport. Moreover, the bimetallic sulfide CuCo_2_S_4_ nanoparticles can provide rich adsorption sites for anchoring LiPSs and improve the conversion kinetics of LiPSs. Thus, the S/CuCo_2_S_4_-1/CNT cathode can achieve a first discharge capacity of 1104.6 ± 4.3 mAh g^−1^ at 0.2 C with a coulombic efficiency close to 100%. After 100 cycles, the discharge specific capacity can maintain 1021 mAh g^−1^. In addition, a reversible capacity of 627 mAh g^−1^ is demonstrated at 1 C after 500 cycles. This work provides a promising strategy for the design of a bimetallic sulfide-CNT network as a sulfur host for Li-S batteries.

## Data Availability

Data are contained within the article.

## Supplementary Materials

The following supporting information can be downloaded at: https://www.mdpi.com/article/10.3390/nano12183104/s1; Figure S1: SEM images of (a) CuCo_2_S_4_-0.5/CNT, (b) CuCo_2_S_4_-1/CNT and (c) CuCo_2_S_4_-2/CNT; Figure S2: Raman spectrum of CNT; Figure S3: (a) N_2_ adsorption/desorption isotherms and (b) pore size distribution of CuCo_2_S_4_-2/CNT. (c) N_2_ adsorption/desorption isotherms and (d) pore size distribution of CuCo_2_S_4_-0.5/CNT.; Figure S4: XRD patterns of S/CuCo_2_S_4_-1/CNT; Figure S5: The first cycle CV curves of S/CuCo_2_S_4_-1/CNT, S/CuCo_2_S_4_-2/CNT and S/CuCo_2_S_4_-0.5/CNT cathodes at the scan rate of 0.1 mV s^−^^1^; Figure S6: Cycling performances of S/CuCo_2_S_4_-1/CNT cathodes at 0.2 C; Figure S7: Charge-discharge curves at 0.2 C of (a) S/CuCo_2_S_4_-2/CNT, (b) S/CuCo_2_S_4_-0.5/CNT and (c) S/CuCo_2_S_4_ cathodes; Figure S8: Charge/discharge voltage profiles at 0.2 C, 0.5 C, 1 C, 2 C and 3 C of (a) S/CuCo_2_S_4_-2/CNT, (b) S/CuCo_2_S_4_-0.5/CNT and (c) S/CuCo_2_S_4_ cathodes. Figure S9: CV curves of symmetric cells with CuCo_2_S_4_-1/CNT electrodes at 6 mV s^−1^.

## References

[1] Zhou G.M., Chen H., Cui Y. (2022). Formulating energy density for designing practical lithium-sulfur batteries. Nat. Energy.

[2] Shao Q.J., Wu Z.S., Chen J. (2019). Two-dimensional materials for advanced Li-S batteries. Energy Storage Mater..

[3] Pei F., Dai S.Q., Guo B.F., Xie H., Zhao C.W., Cui J.Q., Fang X.L., Chen C.M., Zheng N.F. (2021). Titanium-oxo cluster reinforced gel polymer electrolyte enabling lithium-sulfur batteries with high gravimetric energy densities. Energy Environ. Sci..

[4] Li Y.J., Gao T.T., Ni D.Y., Zhou Y., Yousaf M., Guo Z.Q., Zhou J.H., Zhou P., Wang Q., Guo S.J. (2022). Two birds with one stone: Interfacial engineering of multifunctional janus separator for lithium–sulfur batteries. Adv. Mater..

[5] Song X.Q., Tian D., Qiu Y., Sun X., Jiang B., Zhao C.H., Zhang Y., Fan L.S., Zhang N.Q. (2022). Accelerating sulfur redox reactions by topological insulator Bi_2_Te_3_ for high-performance li-s batteries. Adv. Funct. Mater..

[6] Shen J.D., Xu X.J., Liu J., Liu Z.B., Li F.K., Hu R.Z., Liu J.W., Hou X.H., Feng Y.Z., Yu Y. (2019). Mechanistic understanding of metal phosphide host for sulfur cathode in high-energy-density lithium-sulfur batteries. ACS Nano.

[7] Luo D., Li G.R., Deng Y.P., Zhang Z., Li J.D., Liang R.L., Li M., Jiang Y., Zhang W.W., Liu Y.S. (2019). Synergistic engineering of defects and architecture in binary metal chalcogenide toward fast and reliable lithium-sulfur batteries. Adv. Energy Mater..

[8] Song J.X., Yu Z.X., Gordin M.L., Wang D.H. (2016). Advanced sulfur cathode enabled by highly crumpled nitrogen-doped graphene sheets for high-energy-density lithium-sulfur batteries. Nano Lett..

[9] Zhang Y.Z., Wu Z.Z., Pan G.L., Liu S., Gao X.P. (2017). Microporous carbon polyhedrons encapsulated polyacrylonitrile nanofibers as sulfur immobilizer for lithium-sulfur battery. ACS Appl. Mater. Interfaces.

[10] Zhang H., Zhao W.Q., Zou M.C., Wang Y.S., Chen Y.J., Xu L., Wu H.S., Cao A.Y. (2018). 3D, Mutually embedded MOF@carbon nanotube hybrid networks for high-performance lithium-sulfur batteries. Adv. Energy Mater..

[11] Zhu Q.Z., Zhao Q., An Y.B., Anasori B., Wang H.R., Xu B. (2017). Ultra-microporous carbons encapsulate small sulfur molecules for high performance lithium-sulfur battery. Nano Energy.

[12] Chung S.H., Han P., Singhal R., Kalra V., Manthiram A. (2015). Electrochemically stable rechargeable lithium-sulfur batteries with a microporous carbon nanofiber filter for polysulfide. Adv. Energy Mater..

[13] Lv X.X., Lei T.Y., Wang B.J., Chen W., Jiao Y., Hu Y., Yan Y.C., Huang J.W., Chu J.W., Yan C.Y. (2019). An efficient separator with low li-ion diffusion energy barrier resolving feeble conductivity for practical lithium-sulfur batteries. Adv. Energy Mater..

[14] He L., Yang D., Zhao H.N., Wei L.Y., Wang D.S., Wang Y.Z., Chen G., Wei Y.J. (2022). Bipolar CoSe_2_ nanocrystals embedded in porous carbon nanocages as an efficient electrocatalyst for Li-S batteries. Chem. Eng. J..

[15] Huang Y.C., Hsiang H.I., Chung S.H. (2022). Investigation and Design of High-Loading Sulfur Cathodes with a High-Performance Polysulfide Adsorbent for Electrochemically Stable Lithium–Sulfur Batteries. ACS Sustain. Chem. Eng..

[16] Marangon D.V., Scaduti E., Vinci V.F., Hassoun P.J. (2022). Scalable Composites Benefiting from Transition-Metal Oxides as Cathode Materials for Efficient Lithium-Sulfur Batteries. ChemElectroChem.

[17] Cheng C.S., Chung S.H. (2022). Rational Design of High-Performance Nickel-Sulfur Nanocomposites by the Electroless Plating Method for Electrochemical Lithium-Sulfur Battery Cathodes. Batteri. Supercaps.

[18] Li M.C., Liu Z., Tan L., Zhou Q.Y., Zhang J.J., Hou P.P., Jin X.J., Lv T.B., Zhao Z.Q., Zeng Z.L. (2022). Fabrication of Cubic and Porous Carbon Cages with In-Situ-Grown Carbon Nanotube Networks and Cobalt Phosphide for High-Capacity and Stable Lithium–Sulfur Batteries. ACS Sustain. Chem. Eng..

[19] Wang C.L., Sun L.S., Li K., Wu Z.J., Zhang F.F., Wang L.M. (2020). Unravel the catalytic effect of two-dimensional metal sulfides on polysulfide conversions for lithium–sulfur batteries. ACS Appl. Mater. Interfaces.

[20] Hosseini S.M., Varzi A., Ito S., Aihar Y., Passerini S. (2020). High loading CuS-based cathodes for all-solid-state lithium sulfur batteries with enhanced volumetric capacity. Energy Storage Mater..

[21] Li W.L., Qian J., Zhao T., Ye Y.S., Xing Y., Huang Y.X., Wei L., Zhang N.X., Chen N., Li L. (2019). Boosting high-rate Li-S batteries by an MOF-derived catalytic electrode with a layer-by-layer structure. Adv. Sci..

[22] Ai G., Hu Q.Q., Zhang L., Dai K.H., Wang J., Xu Z.J., Huang Y., Zhang B., Li D., Zhang T. (2019). Investigation of the nanocrystal CoS_2_ embedded in 3D honeycomb-like graphitic carbon with a synergistic effect for high-performance lithium sulfur batteries. ACS Appl. Mater. Interfaces.

[23] Liang K., Marcus K., Zhang S.F., Zhou L., Li Y.L., De Oliveira S.T., Orlovskaya N., Sohn Y.H., Yang Y. (2017). NiS_2_/FeS holey film as freestanding electrode for high-performance lithium battery. Adv. Energy Mater..

[24] Liu Y.G., Wang W.K., Wang A.B., Jin Z.Q., Zhao H.L., Yang Y.S. (2017). A polysulfide reduction accelerator-NiS_2_-modified sulfurized polyacrylonitrile as a high performance cathode material for lithium-sulfur batteries. J. Mater. Chem. A.

[25] Zhang H., Zou M.C., Zhao W.Q., Wang Y.S., Chen Y.J., Wu Y.Z., Dai L.X., Cao A.Y. (2019). Highly dispersed catalytic Co_3_S_4_ among a hierarchical carbon nanostructure for high-rate and long-life lithium-sulfur batteries. ACS Nano.

[26] Xu H.H., Manthiram A. (2017). Hollow cobalt sulfide polyhedra-enabled long-life, high areal-capacity lithium-sulfur batteries. Nano Energy.

[27] Czioska S., Wang J.Y., Teng X., Chen Z.F. (2018). Hierarchically structured CuCo_2_S_4_ nanowire arrays as efficient bifunctional electrocatalyst for overall water splitting. ACS Sustain. Chem. Eng..

[28] Huang Y.G., Lv D.J., Zhang Z.J., Ding Y.J., Lai F.Y., Wu Q., Wang H.Q., Li Q.Y., Cai Y.Z., Ma Z.L. (2020). Co-Fe bimetallic sulfide with robust chemical adsorption and catalytic activity for polysulfides in lithium-sulfur batteries. Chem. Eng. J..

[29] Lu X.L., Zhang Q.F., Wang J., Chen S.H., Ge J.M., Liu Z.M., Wang L.L., Ding H.B., Gong D.C., Yang H.G. (2019). High performance bimetal sulfides for lithium-sulfur batteries. Chem. Eng. J..

[30] Gao Y.Y., Qin Y.B., Zhang M., Xu L.H., Yang Z.C., Xu Z.L., Wang Y., Men M. (2022). Revealing the role of oxygen-containing functional groups on graphene oxide for the highly efficient adsorption of thorium ions. J. Hazard. Mater..

[31] Hasanvandian F., Salmasi M.Z., Moradi M., Saei S.F., Kakavandi B., Setayesh S.R. (2022). Enhanced spatially coupling heterojunction assembled from CuCo_2_S_4_ yolk-shell hollow sphere capsulated by Bi-modified TiO_2_ for highly efficient CO_2_ photoreduction. Chem. Eng. J..

[32] Liu S.D., Kang L., Hu J.S., Jung E., Henzie J., Alowasheeir A., Zhang J., Miao L., Yamauchi Y., Jun S.C. (2022). Realizing superior redox kinetics of hollow bimetallic sulfide nanoarchitectures by defect-induced manipulation toward flexible solid-state supercapacitors. Small.

[33] Wang Z.F., Fei P.Y., Xiong H.Q., Qin C.L., Zhao W.M., Liu X.Z. (2017). CoFe_2_O_4_ nanoplates synthesized by dealloying method as high performance Li-ion battery anodes. Electrochim. Acta.

[34] Wang Z.F., Zhang Y.S., Xiong H.Q., Qin C.L., Zhao W.M., Liu X.Z. (2018). Yucca fern shaped CuO nanowires on Cu foam for remitting capacity fading of Li-ion battery anodes. Sci. Rep..

[35] Wang X.Z., Liu S., Zhang H., Zhang S.S., Meng G., Liu Q., Sun Z.Y., Luo J., Liu X.J. (2022). Polycrystalline SnS_x_ nanofilm enables CO_2_ electroreduction to formate with high current density. Chem. Commun..

[36] An C.H., Kang W., Deng Q.B., Hu N. (2022). Pt and Te codoped ultrathin MoS_2_ nanosheets for enhanced hydrogen evolution reaction with wide pH range. Rare Met..

[37] Pan Z.H., Chen H., Yang J., Ma Y.Y., Zhang Q.C., Kou Z.K., Ding X.Y., Pang Y.J., Zhang L., Gu Q.L. (2019). CuCo_2_S_4_ nanosheets@n-doped carbon nanofibers by sulfurization at room temperature as bifunctional electrocatalysts in flexible quasi-solid-state Zn-Air batteries. Adv. Sci..

[38] Wang Z.F., Zhang X.M., Liu X.L., Zhang W.Q., Zhang Y.G., Li Y.Y., Qin C.L., Zhao W.M., Bakenov Z. (2020). Dual-network nanoporous NiFe_2_O_4_/NiO composites for high performance Li-ion battery anodes. Chem. Eng. J..

[39] Zhang Y.G., Liu J.B., Wang J.Y., Zhao Y., Luo D., Yu A.P., Wang X., Chen Z.W. (2021). Engineering oversaturated Fe-N_5_ multi-functional catalytic sites for durable lithium-sulfur batteries. Angew. Chem. Int. Ed..

[40] Fang D.L., Sun P., Huang S.Z., Shang Y., Li X.L., Yan D., Von Lim Y., Su C.Y., Su B.J., Juang J.Y. (2022). An exfoliation-evaporation strategy to regulate N coordination number of Co single-atom catalysts for high-performance lithium-sulfur batteries. ACS Mater. Lett..

[41] Fu Y.Z., Su Y.S., Manthiram A. (2013). Highly reversible lithium/dissolved polysulfide batteries with carbon nanotube electrodes. Angew. Chem. Int. Ed..

[42] Song Y.L., Wang Z.F., Yan Y.J., Zhao W.M., Zhumabay B. (2021). NiCo_2_S_4_ nanoparticles embedded in nitrogen-doped carbon nanotubes networks as effective sulfur carriers for advanced Lithium–Sulfur batteries. Microporous Mesoporous Mat..

[43] Li Y.J., Wu J.B., Zhang B., Wang W.Y., Zhang G.Q., Seh Z.W., Zhang N., Sun J., Huang L., Jiang J.J. (2020). Fast conversion and controlled deposition of lithium (poly)sulfides in lithium-sulfur batteries using high-loading cobalt single atoms. Energy Storage Mater..

[44] Wang Z.F., Zhang X.M., Liu X.L., Zhang Y.G., Zhao W.M., Li Y.Y., Qin C.L., Bakenovc Z. (2020). High specific surface area bimodal porous carbon derived from biomass reed flowers for high performance lithium-sulfur batteries. J. Colloid Interface Sci..

[45] Jiang W., Dong L.L., Liu S.H., Zhao S.S., Han K.R., Zhang W.M., Pan K.F., Zhang L.P. (2022). NiFe_2_O_4_/ketjen black composites as efficient membrane separators to suppress the shuttle effect for long-life lithium-sulfur batteries. Nanomaterials.

[46] Yan Y.J., Chen Y.X., Wang Z.F., Qin C.L., Bakenov Z., Zhao Y. (2021). Flower-like Ni_3_S_2_ hollow microspheres as superior sulfur hosts for lithium-sulfur batteries. Microporous Mesoporous Mat..

[47] Li G.R., Qiu W.L., Gao W.J., Zhu Y.J., Zhang X.M., Li H.Y., Zhang Y.G., Wang X., Chen Z.W. (2022). Finely-dispersed Ni_2_Co nanoalloys on flower-like graphene microassembly empowering a bi-service matrix for superior lithium-sulfur electrochemistry. Adv. Funct. Mater..

[48] Zhou S.Y., Yang S., Ding X.W., Lai Y.C., Nie H.G., Zhang Y.G., Chan D., Duan H., Huang S.M., Yang Z. (2020). Dual-regulation strategy to improve anchoring and conversion of polysulfides in lithium-sulfur batteries. ACS Nano.

