# Supplementary material for: CuCo2S4 Nanoparticles Embedded in Carbon Nanotube Networks as Sulfur Hosts for High Performance Lithium-Sulfur Batteries
Source: Nanomaterials (Basel). 2022 Sep 7;12(18):3104. doi: 10.3390/nano12183104 (PMC9501008; doi:10.3390/nano12183104)
Supplement: Supplementary file 1 [file nanomaterials-12-03104-s001.zip › nanomaterials-1889630-supplementary.pdf]

Supporting Information

$CuCo_2S_4$ Nanoparticles Embedded in Carbon Nanotube Networks as Sulfur Hosts for High Performance Lithium-Sulfur Batteries

Hongying Wang [1,2,†], Yanli Song [1,†], Yanming Zhao [1], Yan Zhao [1,*] and Zhifeng Wang [1,2,*]

[1] School of Materials Science and Engineering, Hebei University of Technology, Tianjin 300401, China
[2] Key Laboratory for New Type of Functional Materials in Hebei Province, Hebei University of Technology, Tianjin 300401, China
* Correspondence: yanzhao1984@hebut.edu.cn (Y.Z.); wangzf@hebut.edu.cn (Z.W.); Tel.: +86-22-6020-4060 (Y.Z.); +86-22-6020-2006 (Z.W.)
† These authors contributed equally to this work.

S0 Materials and Methods

Fabrication of S/$CuCo_2S_4$/CNT and S/$CuCo_2S_4$ composites

The as-prepared $CuCo_2S_4$-1/CNT, $CuCo_2S_4$-2/CNT, $CuCo_2S_4$-0.5/CNT and $CuCo_2S_4$ were mixed and ground with sulfur in the ratio of 1:4 by weight, and then heated to 155 °C under argon for 12 h to obtain S/$CuCo_2S_4$-1/CNT, S/$CuCo_2S_4$-2/CNT, S/$CuCo_2S_4$-0.5/CNT and S/$CuCo_2S_4$ composites.

Preparation of Li_2S_6 solution

Li_2S and sulfur powder were dissolved in lithium-sulfur electrolyte in a molar ratio of 1:5. The prepared solution was stirred at 80°C for 12 h.

Material characterization

The morphology of the samples was obtained through scanning electron microscope (SEM, JSM-7100F) and transmission electron microscope (TEM, JEM-2100F). The X-ray diffraction (XRD, D8 Discover, Bruker Inc.) was applied to examine crystallinity and composition of the prepared materials. Raman spectra were implemented on a Raman microscope (DXR 2Xi) to get the molecular structure of the sample. Thermogravimetric analysis (TGA) was researched under Ar with a heating rate of 10 °C min^{-1} to confirm the weight ratio of sulfur in samples. The N_2 adsorption/desorption isotherm was recorded by V-Sorb 2800P analyzer to obtain the pore size and specific surface area of samples. X-ray photoelectron spectrometry (XPS) was operated with a K-α 1063 XPS analyzer. Ultraviolet-visible (UV-Vis) absorption spectrum of Li_2S_6 was measured with Lambda 25 spectrophotometer (PerkinElmer) in the spectral range of 250–800 nm.

Electrochemical measurements

In order to test electrochemical performance of different electrodes, the working electrodes were prepared by mixing of S/$CuCo_2S_4$-1/CNT (S/$CuCo_2S_4$-2/CNT, S/$CuCo_2S_4$-0.5/CNT and S/$CuCo_2S_4$) composites with PVDF and carbon black with a mass ratio is 8:1:1 in NMP. Then the slurry was covered on the carbon coated aluminum foil, and dried at 60 °C. The sulfur electrodes were controlled to be with a sulfur loading of ~1.2 mg cm^{-2}. The test cells in CR2032 coin-type configuration were assembled in the argon-filled glove box. The electrolyte was 1.0 M of LiTFISI and 1.0 wt% $LiNO_3$ additives in a mixture of equal volumes of DOL and DME, while lithium foil served as both counter and reference electrode. The drop adding amount of electrolyte was 15 μL. Each type was prepared for three samples. The electrochemical performance of assembled cells was analyzed on the NEWARE BTS-4000 testing system. The cyclic voltammetry (CV) measurement of the cells was performed with the CHI660E at a scanning rate of 0.1 mV s^{-1}. Electrochemical impedance spectroscopy (EIS) was measured in a frequency range from 0.01 Hz to 100 kHz.

Symmetric cells measurement

The symmetrical cell electrodes were prepared by mixing the electrode material with polyvinylidene fluoride (PVDF) in a mass ratio of 9:1 in N-Methyl-2-Pyrrolidone (NMP) solvent. Then the slurry was coated on the carbon cloth and dried at 60°C in a vacuum oven. The symmetrical CR2032 coin cells were assembled using the Li_2S_6 electrolyte (50 μL). The CHI660E was utilized to test the CV data in a working potential range of -1.5–1.5 V with a scanning rate of 6 mV s^{-1}. The EIS was measured in a frequency range from 0.01 Hz to 100 kHz.

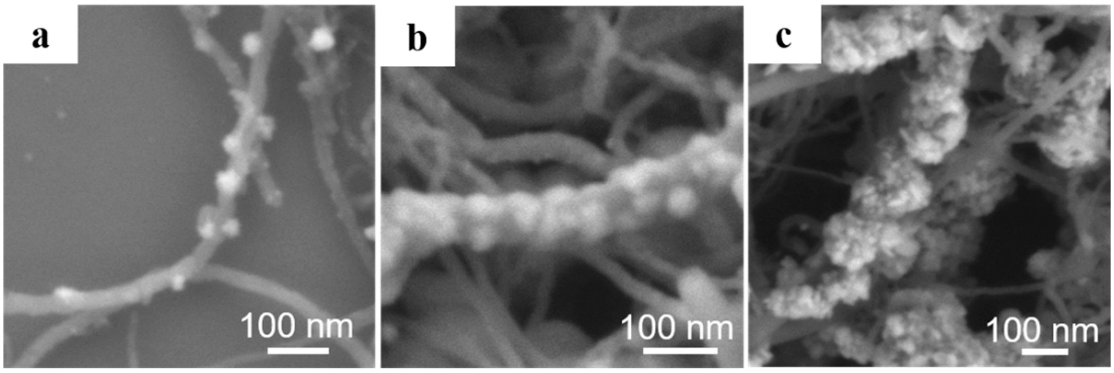

Figure S1. SEM images of (a) $CuCo_2S_4$-0.5/CNT, (b) $CuCo_2S_4$-1/CNT and (c) $CuCo_2S_4$-2/CNT.

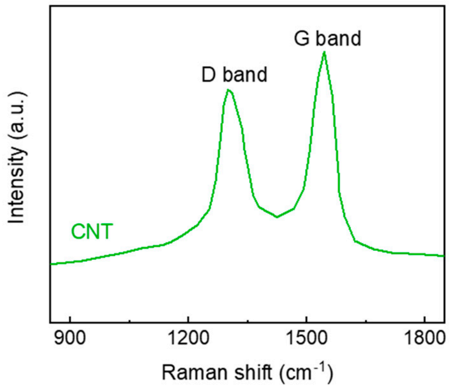

Figure S2. Raman spectrum of CNT.

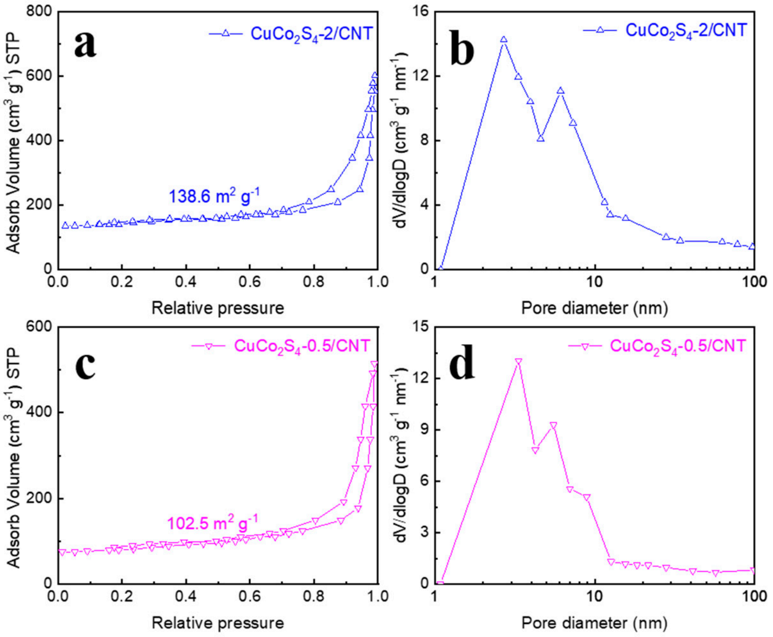

Figure S3. (a) N_2 adsorption/desorption isotherms and (b) pore size distribution of $CuCo_2S_4$-2/CNT. (c) N_2 adsorption/desorption isotherms and (d) pore size distribution of $CuCo_2S_4$-0.5/CNT.

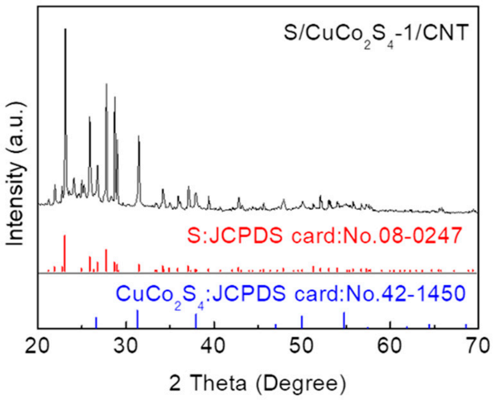

Figure S4. XRD patterns of S/CuCo$_2$S$_4$-1/CNT.

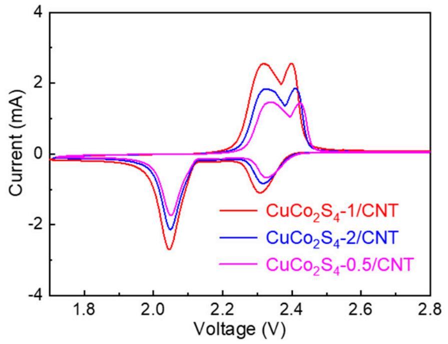

Figure S5. The first cycle CV curves of S/CuCo$_2$S$_4$-1/CNT, S/CuCo$_2$S$_4$-2/CNT and S/CuCo$_2$S$_4$-0.5/CNT cathodes at the scan rate of 0.1 mV s^{-1}.

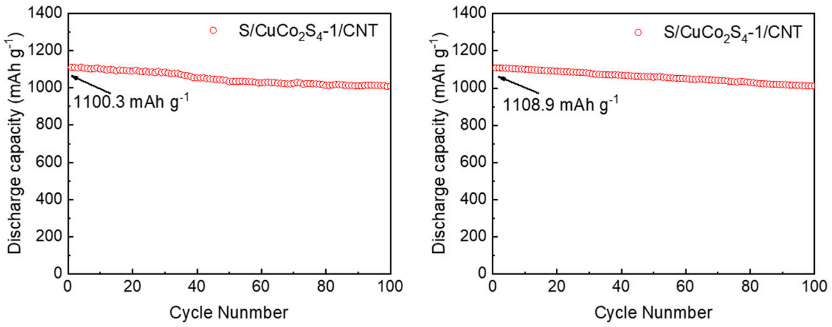

Figure S6. Cycling performances of S/CuCo$_2$S$_4$-1/CNT cathodes at 0.2 C.

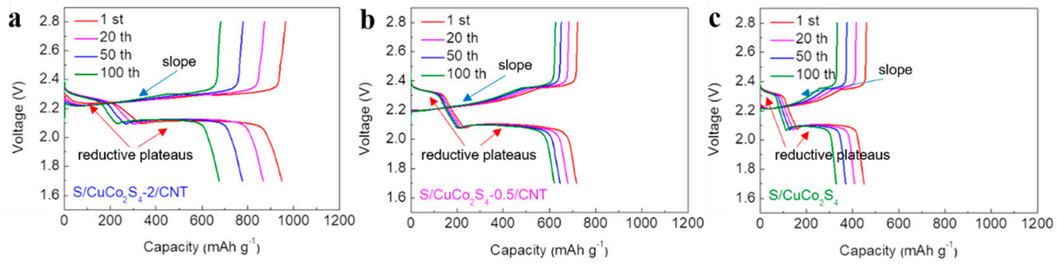

Figure S7. Charge-discharge curves at 0.2 C of (a) S/CuCo$_2$S$_4$-2/CNT, (b) S/CuCo$_2$S$_4$-0.5/CNT and (c) S/CuCo$_2$S$_4$ cathodes.

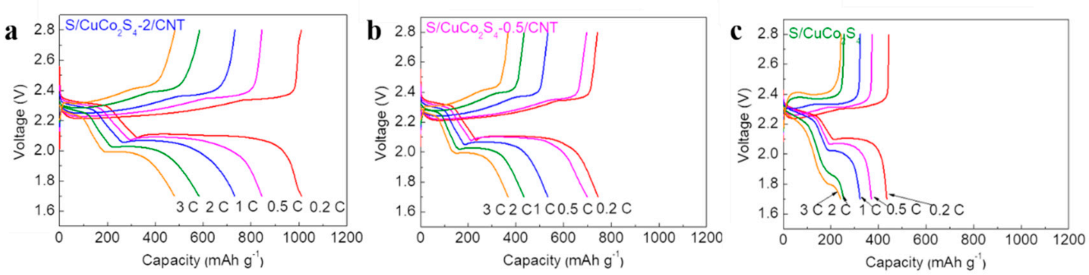

Figure S8. Charge/discharge voltage profiles at 0.2 C, 0.5 C, 1 C, 2 C and 3 C of (a) S/$CuCo_2S_4$-2/CNT, (b) S/$CuCo_2S_4$-0.5/CNT and (c) S/$CuCo_2S_4$ cathodes.

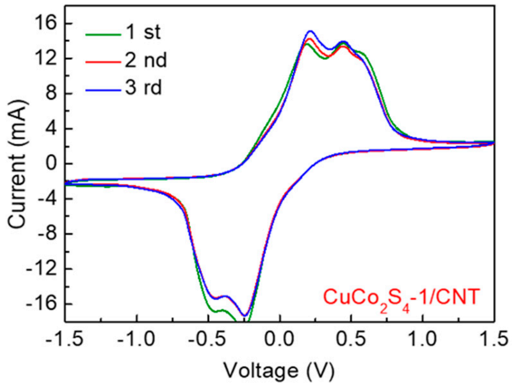

Figure S9. CV curves of symmetric cells with $CuCo_2S_4$-1/CNT electrodes at 6 mV s^{-1}.
